# Understanding Mood of the Crowd with Facial Expressions: Majority Judgment for Evaluation of Statistical Summary Perception

**DOI:** 10.3758/s13414-022-02449-8

**Published:** 2022-03-15

**Authors:** Yoshiyuki Ueda

**Affiliations:** Kokoro Research Center, Kyoto University, 46 Yoshida Shimoadachi-cho, Sakyo, Kyoto, 606-8501 Japan

**Keywords:** Ensemble perception, Statistical summary perception, Majority judgement, Facial expression, Real distinctive face

## Abstract

We intuitively perceive mood or collective information of facial expressions without much effort. Although it is known that statistical summarization occurs even for faces instantaneously, it might be hard to perceive precise summary statistics of facial expressions (i.e., using all of them equally) since recognition of them requires the binding of multiple features of a face. This study assessed which information is extracted from the crowd to understand mood. In a series of experiments, twelve individual faces with happy and neutral expressions (or angry and neutral expressions) were presented simultaneously, and participants reported which expression appeared more frequently. To perform this task correctly, participants must perceive precise distribution of facial expressions in the crowd. If participants could perceive ensembles based on every face instantaneously, expressions presented on more than half of the faces (in a single ensemble/trial) would have been identified as more frequently presented and the just noticeable difference would be small. The results showed that participants did not always report seeing emotional faces more frequently until much more emotional than neutral faces appeared, suggesting that facial expression ensembles were not perceived from all faces. Manipulating the presentation layout revealed that participants’ judgments highly weight only a part of the faces in the center of the crowd regardless of their visual size. Moreover, individual differences in the precision of summary statistical perception were related to visual working memory. Based on these results, this study provides a speculative explanation of summary perception of real distinctive faces. (247 words)

In daily life, we see many faces and read a variety of information from them. For example, we infer the state of others’ emotions or mood based on single face we encounter. Besides that, we can also use collective information of multiple faces to infer these emotions or moods. Supposing that an individual presents a talk, he or she can determine whether the audience is enjoying (or understanding) the talk at a glance, and that a person takes a group photograph, they can instantaneously determine whether the photographic subjects are smiling. This perception does not rely on single face recognition alone. Specifically, collection of facial expressions needs to be accessed when judging whether a group is harmonious or not. We can perceive them intuitively and it does not require much effort.

This ability relates to statistical summary perception (or ensemble perception), in which individuals instantly create statistical summaries (c.f., average and variance) of visually presented items, and is thought to be basis for further cognitive processing such as scene recognition (e.g., Alvarez, 2011; Ariely, 2001; Utochkin, 2015). This perception has been shown with both low-level features, such as orientation, size, luminance, color, and motion direction and speed, and more complicated objects, such as facial identities and facial expressions (de Fockert & Wolfenstein, 2008; Haberman, Brady, & Alvarez, 2015a; Haberman & Whitney, 2007, 2009; for a review, Whitney & Yamanashi-Leib, 2018). In studies conducted by de Fockert and Wolfenstein (2008) and Haberman and Whitney (2007, 2009), the facial ensembles of four faces were examined and showed that participants can perceive the average of them with quite better performance. Subsequent studies presented more than four faces and demonstrated that participants could still perceive face ensemble properties such as averages and variance (Bai et al., 2015; Haberman, Lee, & Whitney, 2015b; Haberman & Whitney, 2010). Whitney & Yamanashi-Leib (2018) mentioned that ensemble can be formed with approximately the square root of the number of presented items both for low-level features and complicated objects.

One of the interesting issues is that ensemble of faces seems to be extracted as well as low-level features. In general, recognizing facial expression is a much more complicated process than relatively low-level features (e.g., Bruce & Young, 1986). According with Ekman and Friesen (1978), facial expression is determined by combinations of facial muscles (described as action units). For example, a happy face is characterized by tightened muscles around the eyes, with the cheeks and corners of the lips raised, whereas an angry face is characterized by lowered, drawn-together eyebrows with tensed and raised eyelids and lip-pressed-against-lip mouth (Ekman & Friesen, 2003). It indicates that recognizing facial expressions require multiple feature-binding. Hence, facial expression ensemble is achieved through summarizing multiple features simultaneously; that is, different processes are required between facial expression ensemble and simple feature ensemble. In accordance with this perspective, Haberman, Brady, and Alvarez (2015a) suggested that the mechanisms of ensemble perception for low-level features and complicated objects might be different from each other. In their experiments, they measured sensitivity to ensembles of low-level features (i.e., orientation and luminance) and faces (i.e., identity and facial expressions) and showed that individual correlations between levels of sensitivity to ensembles of low-level features and individual correlations between faces were strong, respectively, while correlations between levels of sensitivity to ensembles of low-level features and faces were weak.

So far, there remain unclear points concerning how to extract facial expression statistics and how to understand the mood or collective information of faces, although facial expression ensemble itself is obviously achieved. One of them is that judgment in which positive expressions or negative expressions are frequently displayed in the crowd (i.e., good mood or bad mood) is difficult through simple average perception alone. For example, in a case where a few display extremely strong positive expressions and others show slightly negative expressions, the average of their expressions is relatively positive. Therefore, average perception does not necessarily lead to a correct answer to majority judgment considering the number and intensity (see Nagy, Zimmer, Greenlee, & Kovács, 2012). Although counting the items is the best strategy, it is impractical as counting items at a glance is difficult, except for a small number of items (Kaufman et al., 1949). To achieve precise majority estimation, participants require recognizing precise distribution (i.e., the ratios of emotional faces) from the crowds.

Some previous studies have employed a binary choice task, although they did not ask for majority estimation. Yang et al. (2013) asked participants to indicate whether the facial crowd was positive or negative as a whole while presenting happy and angry expressions. Although they did not clearly identify what information the observers should extract (c.f., average or distribution), one concern that has arisen from their results is that summary statistic perception of faces is less precise than has been considered. Fitting to a cumulative Gaussian function, Yang et al. (2013) calculated the point of subjective equality (PSE), which reflected observer’s bias, and the standard deviation (SD), which reflects observer’s precision. Their average PSE was 0.54, meaning that the observer had a 50% chance of judging the crowd as negative when 54% of the faces had an angry expression. This indicated that bias of perception was small. On the other hand, the average SD was 0.49. This value was the same as 0.33 in just noticeable difference (JND)^1^, indicating that participants could respond with more than 75% accuracy when more than 87% of faces showed angry expressions. JND is expected to be close to 0 if ensemble perception is accurate enough.

Therefore, this study sought to determine whether participants could perceive precise statistical summary of facial expressions, and if they could not, what information they extract from the crowding of facial expressions using a majority judgment task. For deciding the general method of this study, we summed up the methods of the previous studies and clarified important points of statistical summary perception of facial expression.

First, studies examining facial expression ensemble often presented morphed faces, which were made by merging multiple faces (e.g., expression of happiness and anger of the same person), to participants as stimuli (de Fockert & Wolfenstein, 2008; Haberman & Whitney, 2007; Haberman & Whitney, 2009). Using morphed faces allows researchers to control physical parameters of features quantitatively (i.e., the degree of raised lip corner). On the other hand, calculating average would come to be easier because all features of a face simultaneously change with the same degree; that is, observers are able to focus one or some of the features to calculate average instead of all of them. Moreover, presenting the same person’s morphed faces enables observers easily to extract differences of facial features among pictures compared with presenting a real different person’s faces. Hence, first, the experiments in this study used distinctive faces and investigated the precision of facial ensemble perception (as Ji et al., 2014; Ji, Chen, et al., 2018a; Ji, Rossi, & Pourtois, 2018b; Yang et al., 2013).

Second, faces were presented with the hair and neck cropped out in some studies (Haberman & Whitney, 2007; Haberman & Whitney, 2009; Ji et al., 2014; Ji, Chen, et al., 2018a; Ji, Rossi, & Pourtois, 2018b). However, in the real world, people first have to extract facial information from a whole face, including the hair and neck (sometimes glasses), and then, calculate facial ensemble; that is, cropped face ensemble is much easier than ensemble perception in reality. Previous studies have shown that judgment of the attractiveness of an individual was influenced by attractiveness of individuals around him/her (called the cheerleader effect) using not cropped faces (Walker & Vul, 2014). This implied that judgment is affected by facial ensemble of real faces but did not directly examine whether people can perceive facial ensemble or not. Thus, second, we presented more realistic faces to participants and investigated whether accurate facial ensemble perception is achieved with them.

Third concerns to the number of faces presented to observers. Some studies required observers to sum up information of four faces and showed that perceived facial expression ensemble was possible (De Fockert & Wolfenstein, 2008; Haberman & Whitney, 2007 and 2009; Ji, Rossi, & Pourtois, 2018b). However, in some everyday life situations (such as those in which individuals are in front of audiences) more than four people are observed simultaneously (as Ji et al., 2014; Ji, Chen, et al., 2018a; Yang et al., 2013). In such a case, ensemble is formed with approximately the square root of the number of presented items (Whitney & Yamanashi-Leib, 2018). Attarha and Moore (2015) stated that complex summaries such as facial average may require an additional step to integrate, producing an information-processing bottleneck. Empirically, Ji, Chen, et al. (2018a) have shown that ensemble representation for multiple facial expressions is capacity limited. This enables the third point, that is, how people achieved facial expression summaries of distribution: Whether they extract information from a limited number of faces or calculate ensemble based on the whole, but less precise facial information (e.g., due to interference with each other) when many faces are presented.

In a series of experiments, 12 faces with either happy or neutral expressions (or angry or neutral expressions) were presented, and participants were asked to judge which expression was presented more frequently in the group. Based on observed responses, we calculated the PSE and JND. If participants instantaneously recognized ensembles of all faces, we would expect them to identify the expression presented on more than half of the faces as the more frequently presented expression (i.e., PSE is 0.5 and JND is small enough). For example, when seven faces with happy expressions and five faces with neutral expressions were presented, participants were expected to identify happiness as the more frequently presented expression. Considering noises, this prediction enables us to assume that the results should form a sigmoid-shape (i.e., cumulative normal distribution) when judgments indicating that faces with emotional expressions were presented more frequently (hereafter, these are called “positive responses”) functioned as the actual proportion of faces with emotional expressions presented (Fig. 1a)^2^. Otherwise, if observers perceive ensembles based on less accurate statistics, the results were expected to be closer to a linear and flat function, indicating larger JND (Fig. 1b).

**Fig. 1:**
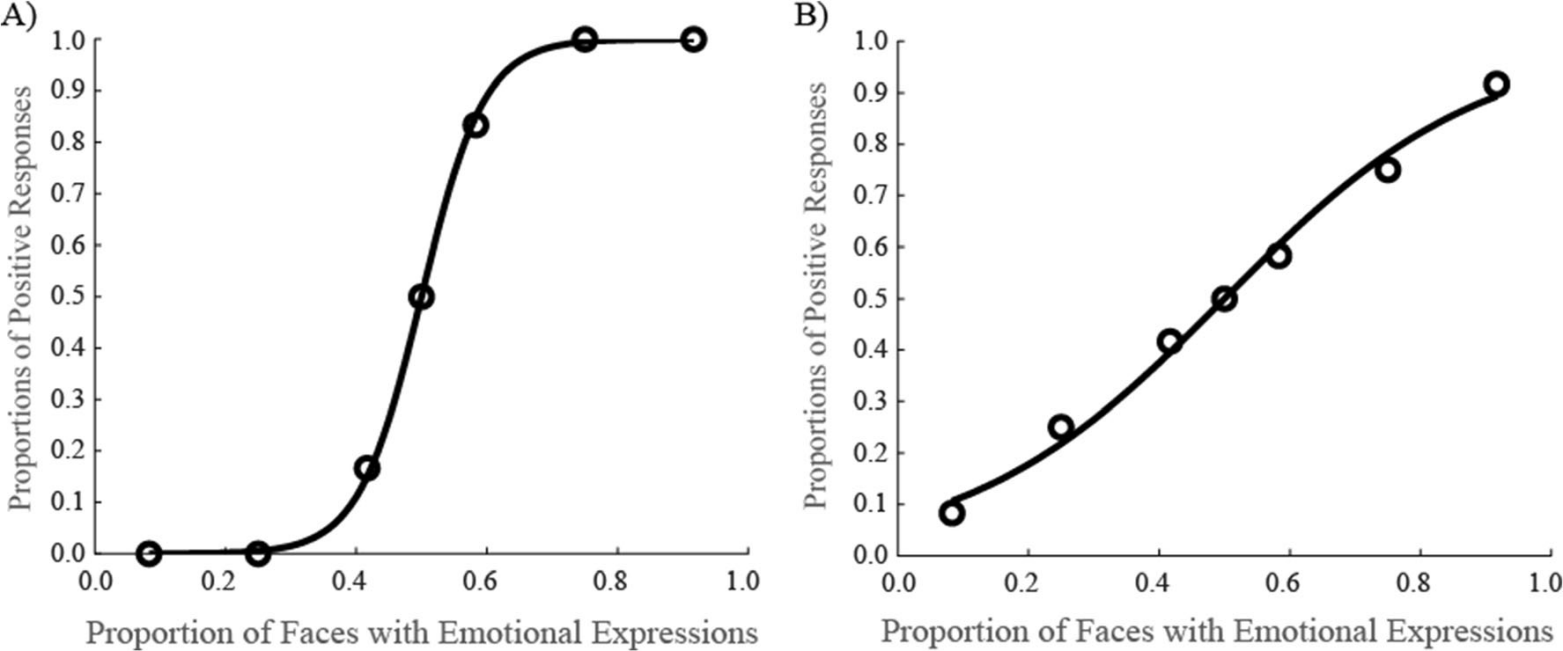
Predictions of the probability that faces with emotional expressions were presented more frequently when participants perceived ensembles with statistics of all faces (**a**) and less of them (**b**)

Experiment 1 examined whether participants could determine which expression was presented more frequently within groups of 12 faces. However, they could not perform this task with precision (i.e., large JND and the results were closer to a linear function rather than a sigmoid-shape). In Experiment 2, the duration of the presentation of the faces was extended, and the results were consistent with those of Experiment 1. In Experiment 3, the location in which faces were presented was changed from the central visual field to the peripheral visual field, but the results were consistent with those of the previous experiments. Experiments 4–6 examined the possibility that judgments regarding facial expressions were based on some faces, and the results indicated that perception of ensembles of facial expressions was based on a small number of faces rather than small areas. Experiment 7 showed that the results were the same even when the hair and neck were cropped out. Experiment 8 showed the relationships between the number of faces which were used for majority estimation and visual short-term memory capacity. Taken together, the results suggest that although participants could roughly perceive facial expression ensemble regardless of the hair and neck, their estimation was not based on all presented faces when many distinctive real faces were presented briefly.

## Experimental Design

### Participants

Eighteen students from Kyoto University were recruited for Experiments 1–7. The sample size was determined based on a calculation of the required sample size with 1 – β = .80 and effect size *f* = .25 using G*Power (Faul et al., 2007; Faul et al., 2009) before Experiment 1. Although the required sample size was 17, we decided to collect 18 participants considering the order of counterbalance. After the first experiment, post hoc analysis showed enough power (1 – β were more than .93), therefore we used the same sample size across experiments. However, one participant, from Experiments 3 and 7, respectively, was excluded from the analysis because they misunderstood the task. For Experiment 8, 24 students were recruited to conduct correlation analyses, in which the effect size should be high (ρ = 0.5) based on indication of relationships between working memory and ensemble perception. All participants participated only one of experiments and did not participate in more than one experiment. All participants had normal or corrected-to-normal vision and were naïve to the purpose of the experiments. They received information regarding the study purpose, methodology, and risks; their right to withdraw; the durations of the experiments; the handling of individual information; and the voluntary nature of participation, and provided informed consent prior to initiation of the experiments. The internal review board of Kyoto University approved the procedures.

### Stimuli and Apparatus

Experiments were conducted using a Windows operating system and MATLAB (MathWorks) with the Psychophysics Toolbox (Brainard, 1997; Pelli, 1997; http://psychtoolbox.org/). Stimuli were presented on a cathode ray tube monitor 75 cm from participants’ heads.

In all the experiments, the stimuli were colored photographs of the faces of 44 models (22 men and 22 women) with happy, angry, or neutral facial expressions (in sum, 132 photographs) from the Kokoro Research Center (KRC) facial expression database (Ueda et al., 2019). Thirty undergraduate and graduate students from Kyoto University rated each photo of the database in terms of the emotions happiness and anger on a 7-point scale (1 = very weak, 7 = very intense). The mean intensity ratings for each emotion concerning faces used in this study were as follows: happiness = 4.56 (SD 0.59), anger = 4.42 (SD 0.40). There was no difference between the intensities of happy and angry faces, *t*(43) = 1.35, *p* = .18, *r* = .20. The participants did not recognize both happiness and anger emotions from photos with a neutral expression (rating was < 2.36).

**Fig. 2:**
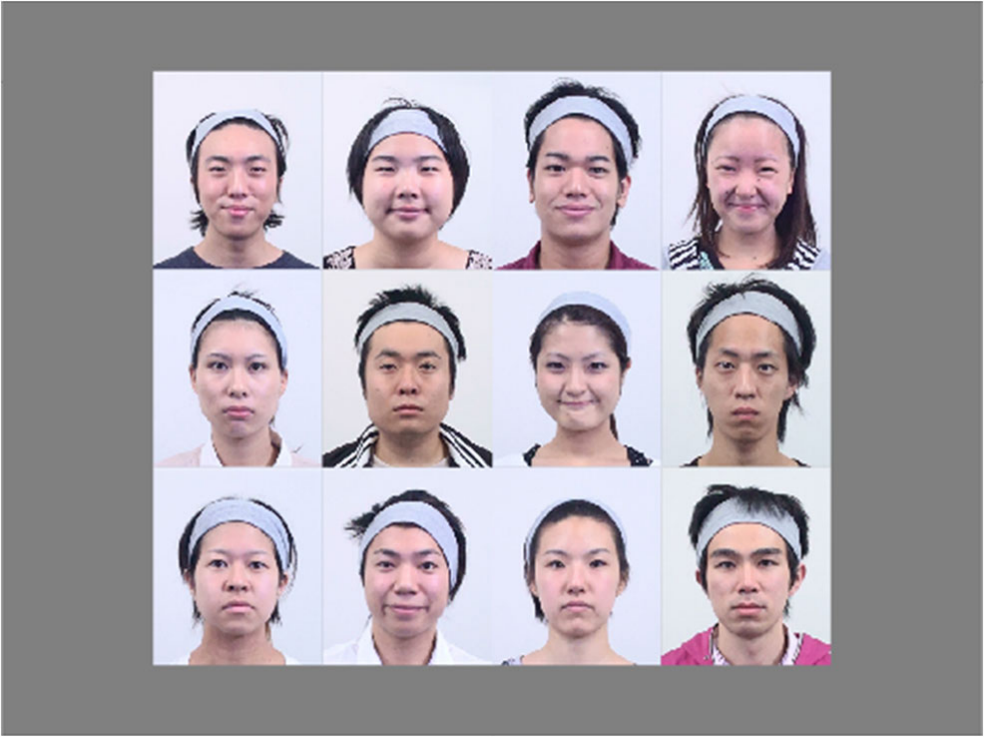
An example of stimuli used in this study. In each trial, twelve faces were presented in a 4 × 3 matrix at the center of the monitor. Faces were presented in color

Each photo included not only the face but also the hair and neck, although a hairband was worn to prevent the faces from being occluded by hair. The photographs were 4.0° wide × 4.7° high in Experiments 1–4, 6, and 8, and 2.0° wide × 2.4° high in Experiment 5. In Experiment 7, the hair and the neck were cropped out. In each trial, twelve faces were presented within a 4 × 3 matrix over an area 15.9° wide × 13.9° high (8.0° wide × 7.1° high in Experiment 6), at the center of the monitor (Fig. 2).

## Experiment 1

To examine precision with facial expression ensembles, participants were asked to indicate which of two facial expressions was presented more frequently. If participants’ correct judgments were based on ensemble information with distribution of all facial expressions, we would expect them to indicate that faces with happy or angry expressions were presented more frequently, relative to neutral faces, when more than half of the faces presented had emotional expressions. Similarly, participants would indicate that faces with neutral expressions were presented more frequently, relative to faces with emotional expressions, when less than half of the faces presented had emotional expressions (see Fig. 1a).

### Procedure

Participants were asked to determine which of two facial expressions (neutral and either happy or angry) was presented more frequently within groups of 12 faces. In each trial, 12 faces were presented simultaneously for 500 ms. Thereafter, participants pressed the “1” or “3” key on the numeric keypad to indicate whether neutral or emotional faces were presented more frequently (key-to-expression correspondence was counter-balanced across participants). No feedback was provided concerning correct responses.

The experiment included two sessions: happy and neutral faces were presented in one session, and angry and neutral faces were presented in another session. The sessions included seven conditions, in which one, three, five, six, seven, nine, or 11 of the 12 presented faces showed emotional expressions, and each condition was presented 40 times. Therefore, each session included 280 trials, and each participant completed 560 trials. Participants also completed 10 practice trials prior to each session. The order of sessions was counterbalanced across participants, and the order of conditions was randomized within participants.

### Results

**Fig. 3:**
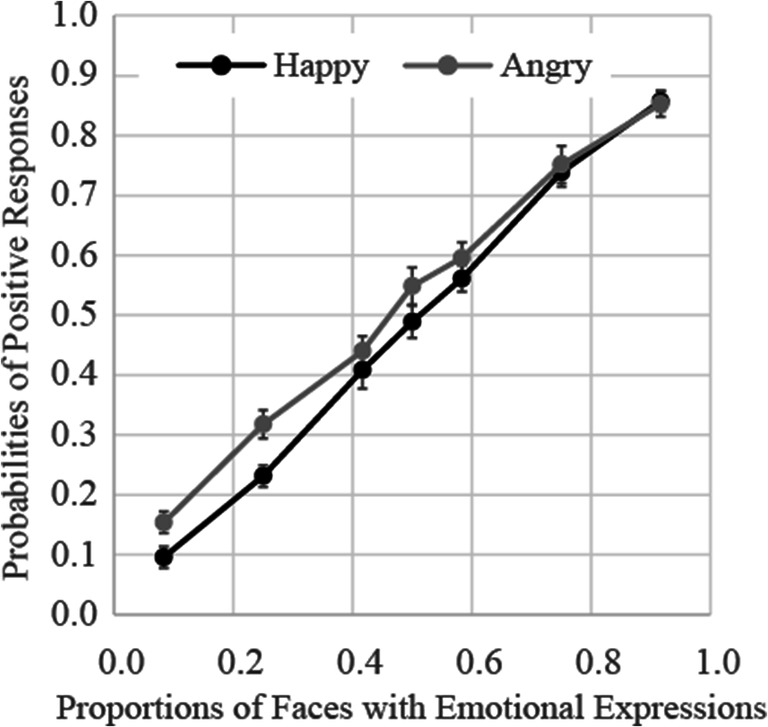
Probabilities of judgments indicating that faces with emotional expressions were presented more frequently in Experiment 1. Error bars represent standard errors

The results of Experiment 1 are presented in Fig. 3. The results of a two-way (facial expression × proportion of emotional stimuli) ANOVA showed that the main effect of facial expression was significant, *F*(1, 17) = 5.72, *p* = .03, ɳ_p_^2^ = .25, indicating that the proportion of angry faces was overestimated relative to that of happy faces. The main effect of proportion of emotional stimuli was also significant, *F*(6, 102) = 326.70, *p* < .0001, ɳ_p_^2^ = .95. Multiple comparisons showed that the proportion of positive responses increased according to increases in the proportion of emotional stimuli. The interaction between facial expression and proportion of emotional stimuli was nonsignificant, *F*(6, 102) = 1.54, *p* = .17, ɳ_p_^2^ = .08.

For each participant, the responses were fitted with a cumulative normal distribution and their PSEs and JNDs were averaged, respectively. The results are shown in the rows of Experiment 1 of Table 1. The values obtained by adding and subtracting PSE and JND were 0.76–0.77 and 0.19–0.28, respectively. This implies that 75% of the emotional face must be presented in order for a positive response to occur with a probability of 75%. Also, the appearance of the function in Fig. 3 looks to be close to linear. One-way ANOVA showed no main effects of facial expression, *F*(1, 17) = 2.88, *p* = .11, ɳ_p_^2^ = .14, and *F*(1, 17) = 3.58, *p* = .08, ɳ_p_^2^ = .17, for the PSE and JND, respectively. This indicates that there were no differences in bias and precision between the happy and angry face presentations.

**Table 1:** The Points of Subjective Equalities (PSEs) and Just Noticeable Differences (JNDs) in a Series of Experiments

Experiment	Target Emotion	PSE		JND	
		Mean	(S.E)	Mean	(S.E.)
Experiment 1	Happy	0.52	(0.02)	0.24	(0.01)
	Angry	0.48	(0.03)	0.29	(0.03)
Experiment 2	Happy	0.55	(0.03)	0.23	(0.01)
	Angry	0.49	(0.01)	0.22	(0.01)
Experiment 3	Happy	0.52	(0.04)	0.32	(0.08)
	Angry	0.49	(0.04)	0.26	(0.02)
Experiment 4					
(Dense Condition)	Happy	0.29	(0.03)	0.16	(0.02)
	Angry	0.27	(0.03)	0.21	(0.03)
(Distributed Condition)	Happy	0.54	(0.02)	0.21	(0.01)
	Angry	0.54	(0.03)	0.24	(0.02)
Experiment 5					
(Dense Condition)	Happy	0.35	(0.03)	0.21	(0.03)
	Angry	0.32	(0.03)	0.26	(0.04)
(Distributed Condition)	Happy	0.59	(0.03)	0.24	(0.02)
	Angry	0.57	(0.03)	0.33	(0.05)
Experiment 6					
(Dense Condition)	Happy	0.57	(0.01)	0.12	(0.01)
	Angry	0.54	(0.01)	0.13	(0.01)
(Distributed Condition)	Happy	0.55	(0.03)	0.20	(0.02)
	Angry	0.54	(0.02)	0.20	(0.01)
Experiment 7					
(Dense Condition)	Happy	0.33	(0.03)	0.22	(0.03)
	Angry	0.33	(0.06)	0.28	(0.05)
(Distributed Condition)	Happy	0.59	(0.02)	0.23	(0.02)
	Angry	0.57	(0.03)	0.29	(0.03)
Experiment 8					
(Dense Condition)	Happy	0.34	( 0.03)	0.18	(0.02)
	Angry	0.33	(0.03)	0.22	(0.04)
(Distributed Condition)	Happy	0.59	(0.03)	0.25	(0.03)
	Angry	0.55	(0.02)	0.25	(0.03)

### Discussion

The results of Experiment 1 showed that the probability of positive responses increased in accordance with increases in the proportion of emotional stimuli. If participants had recognized ensembles of all faces instantaneously, the results should have shown a sigmoid shape; however, they look to be linear, suggesting that participants may not perceive distribution of all facial expressions.

Interestingly, the proportion of angry faces was overestimated relative to that of happy faces. One explanation of this result is due to the anger superiority effect, whereby angry faces are detected more rapidly relative to happy or neutral faces, as they signal hostility (e.g., Eastwood et al., 2001; Fox et al., 2000). Therefore, angry faces could have been identified among neutral faces more rapidly relative to happy faces among neutral faces, leading to greater overestimation of angry faces relative to that of happy faces. However, this effect was not observed in the PSE, suggesting that we must considerably investigate its robustness in this paradigm.

## Experiment 2

In Experiment 1, the duration of the presentation of facial expressions was 500 ms. Although this was enough time for the perception of ensembles of both low-level features and faces (e.g., Chong & Treisman, 2003; Haberman & Whitney, 2009), and that of single facial expression (e.g., Hinojosa et al., 2015), it could have been insufficient for this task. Therefore, Experiment 2 replicated Experiment 1 but extended the duration of the presentation from 500 ms to 1,000 ms. If participants recognized distribution including all faces with this presentation duration, we would expect them to indicate that faces with happy or angry expressions were presented more frequently when more than half of the faces presented had emotional expressions, leading to a decrease in JND.

### Procedure

The tasks and procedures in Experiment 2 were the same as those described for Experiment 1, but the duration of the presentation of faces was extended to 1,000 ms.

### Results

**Fig. 4:**
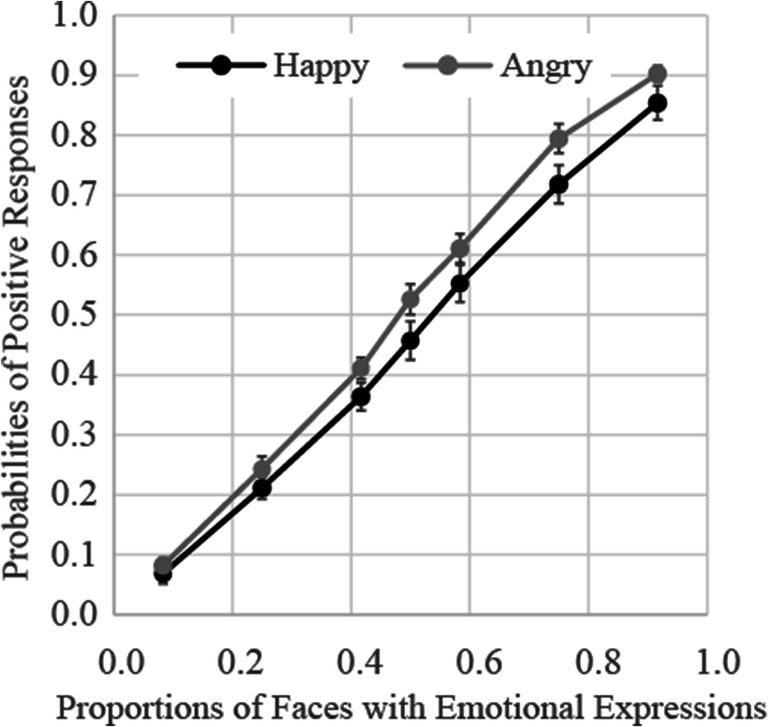
Probabilities of judgments indicating that faces with emotional expressions were presented more frequently in Experiment 2. Error bars represent standard errors

The results of Experiment 2 are presented in Fig. 4. The results of a two-way (facial expression × proportion of emotional stimuli) ANOVA showed that the main effect of facial expression was significant, *F*(1, 17) = 5.56, *p* = .03, ɳ_p_^2^ = .25, indicating that the proportion of angry faces was overestimated relative to that of happy faces. The main effect of proportion of emotional stimuli was also significant, *F*(6, 102) = 386.14, *p* < .0001, ɳ_p_^2^ = .96. Multiple comparisons showed that the probability of judgments indicating that faces with emotional expressions were presented more frequently increased according to increases in the proportion of emotional stimuli. The interaction between facial expression and proportion of emotional stimuli was nonsignificant, *F*(6, 102) = 0.80, *p* = .57, ɳ_p_^2^ = .04.

The average PSEs and JNDs are shown in the rows of Experiment 2 of Table 1. Welch’s two sample *t*-tests showed that there was no difference in the JND of the happy face condition between Experiments 1 and 2, *t*(31.662) = 0.44, *p* = .66, whereas that of the angry face condition was smaller in Experiment 2 than Experiment 1, *t*(22.224) = 2.37, *p* = .03. Moreover, one-way ANOVA showed a main effect of facial expression for the PSE, *F*(1, 17) = 4.70, *p* = .04, ɳ_p_^2^ = .22, but no main effect for the JND, *F*(1, 17) = 2.32, *p* = .15, ɳ_p_^2^ = .12, indicating that positive responses were higher for angry faces than happy faces.

### Discussion

The results of Experiment 2 showed that the probability of positive responses increased in accordance with the proportion of emotional stimuli. The precision of majority judgments for angry faces was better compared with judgments in Experiment 1. However, results for happy faces did not differ from Experiment 1 even for a longer presentation duration.

## Experiment 3

Ensembles play an important role in the perception of scenes outside the focus of attention (Alvarez & Oliva, 2008). Although Wolfe et al. (2015) showed no difference in ensemble perceptions of facial expressions between when participants could use foveal information and when they could not, the presentation of faces at the center of the visual field in previous experiments could have been disadvantageous to the perception of ensembles of facial expressions. Therefore, in Experiment 3, faces were presented only in the peripheral visual field, and participants were asked to determine which of two facial expressions was presented more frequently, as in the task described for Experiments 1 and 2. Participants would be expected to indicate that faces with emotional expressions were presented more frequently when more than half of the faces presented had emotional expressions, if they perceived ensembles of all facial expressions via peripheral vision.

### Procedure

The task completed by participants was the same as that described for Experiments 1 and 2, apart from the locations in which faces were presented. Twelve faces were divided into two groups containing six faces with 2 × 3 matrixes, presented on both the left and right sides of the monitor, with 6.5° from the center of the monitor to the center of each group (Fig. 6a). The probability of appearance of emotional faces (happiness and anger) was the same across groups. The faces were presented for 1,000 ms, as in Experiment 2.

### Results

**Fig. 5:**
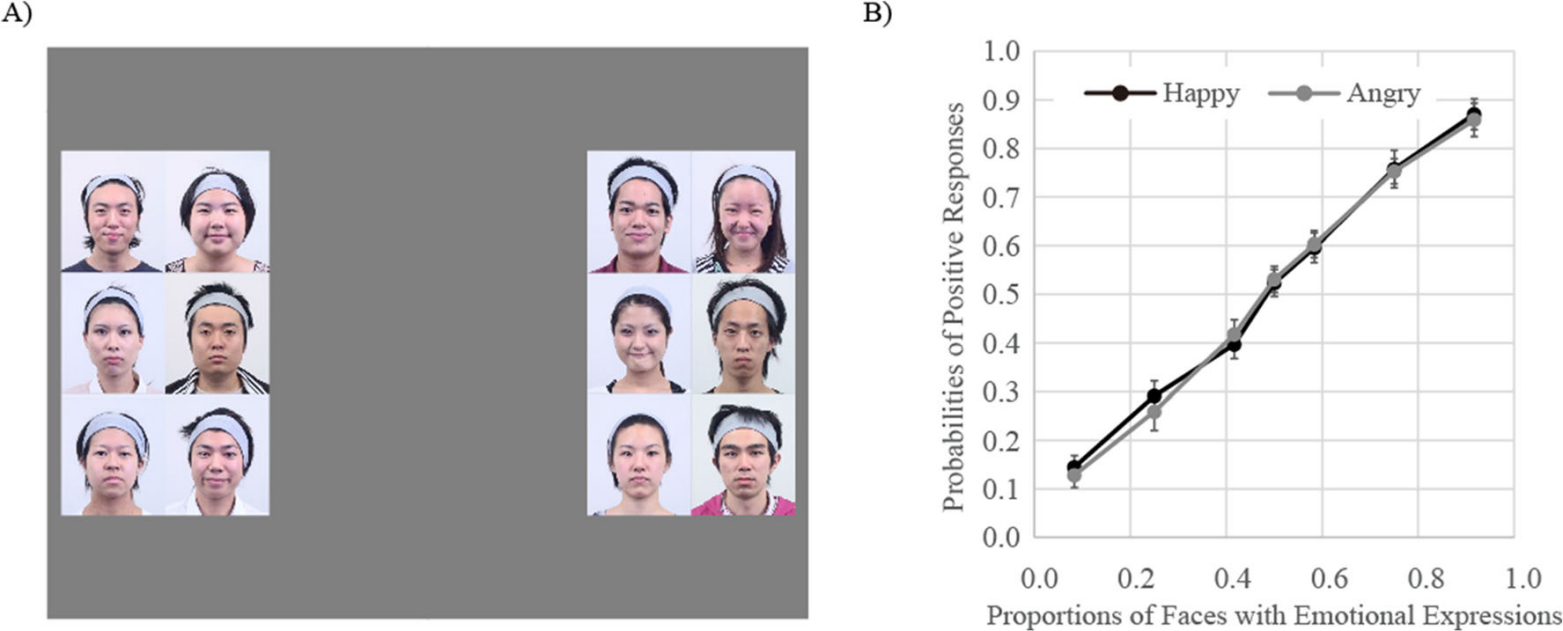
An example of stimuli used in Experiment 3 (**a**), and the probabilities of judgments indicating that faces with emotional expressions were presented more frequently in Experiment 3 (**b**). Error bars represent standard errors

The results of Experiment 3 are shown in Fig. 5b. The results of a two-way (facial expression ×proportion of emotional stimuli) ANOVA showed that the main effect of proportion of emotional stimuli was significant, *F*(6, 96) = 239.36, *p* < .0001, ɳ_p_^2^ = .94. Multiple comparisons showed that probability of judgments indicating that faces with emotional expressions were presented more frequently increased according to increases in the proportion of emotional stimuli. The main effect of facial expression and the interaction between facial expression and proportion of emotional stimuli were nonsignificant, *F*(1, 16) = 0.03, *p* = .86, ɳ_p_^2^ = .00, and *F*(6, 96) = 0.40, *p* = .88, ɳ_p_^2^ = .02.

The average PSEs and JNDs are shown in the rows of Experiment 3 of Table 1. Welch’s two sample *t*-tests showed that the JND in both the happy and angry face conditions were not different from those in Experiment 2, *t*(16.963) = 1.03, *p* = .32, and *t*(23.966) = 1.71, *p* = .10. Moreover, there were no main effects of facial expression for both PSE and JND, *F*s(1, 16) < 0.50, *p*s > .49, ɳ_p_^2^ < .03.

### Discussion

Experiment 3 showed that performance did not change when participants perceived ensembles peripherally. The results were consistent with those of Experiment 2. Note, however, that in Experiment 3, although participants were instructed to look at the center of the display, eye movements were not record, so they may have moved their eyes during the presentation of faces. In that case, the situation would be similar to that of Experiment 1 and Experiment 2, overestimation of the number of angry faces, which was observed in Experiments 1 and 2, was not observed in Experiment 3. The overestimation of angry faces in Experiments 1 and 2 could have occurred because of biases caused by the presentation of angry faces in the center of the visual field.

## Experiment 4

The results of Experiments 1–3 indicated that participants responded in accordance with proportions of faces with emotional expressions within a group of faces, but unable to make a judgment based on accurate distribution including all of the faces. One explanation for this finding could be that participants did not understand distributions of faces with emotional expressions in groups; rather, they summarized the probability of the presentation of faces with emotional expressions in the entire group of faces, and determined which type of face was presented more frequently based on this probability. An alternative explanation is that participants could have perceived ensembles based on a small area or small number of faces (i.e., via subsampling). The expected values for these calculations were the same, and both looked linear functions observed in the previous experiments (see Appendix).

To determine whether participants based their judgments on a small group of faces or the entirety of the faces, Experiment 4 included two presentation patterns: a dense pattern, in which the presentation of faces with emotional expressions was dense at the center of the presentation matrix, and a distributed pattern, in which faces with emotional expressions were presented in random locations (i.e., as in Experiments 1 and 2). If participants based their judgments on probability extracted from the entire ensemble, the results would not differ between the presentation patterns.

### Procedure

**Fig. 6:**
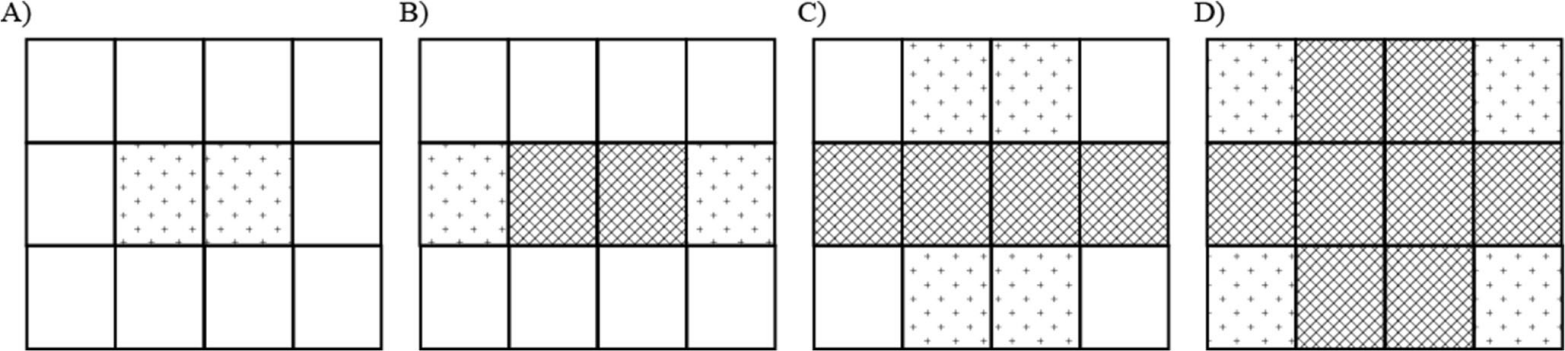
Schema for the dense presentation patterns in Experiments 4 and 5 with one face expressing emotion presented (**a**), three faces with emotional expressions presented (**b**), five, six, and seven faces with emotional expressions presented (**c**), and nine and eleven faces with emotional expressions presented (**d**). The cells with diagonal lines indicate that faces were always presented in each pattern, whereas the cells filled with plus signs indicate that faces were presented randomly in those locations. In the distributed presentation pattern, faces with emotional expressions were located in the matrix randomly, as in Experiments 1 and 2

Participants were required to determine which of two facial expressions was presented more frequently in groups of 12 faces (i.e., as in Experiments 1–3). In the distributed presentation pattern, faces with emotional expressions were presented in random locations in the presentation matrix, as in Experiments 1 and 2. In contrast, in the dense presentation pattern, the presentation of faces with emotional expressions was dense at the center of the presentation matrix (Fig. 6). In the trials that included only one face expressing happiness or anger, it was presented in one of two locations at the center of the presentation matrix. In trials that included three faces with emotional expressions, two of the faces were presented at the center of the matrix, and the other was presented on either the left or right side of the central row of the matrix. In trials that included five, six, or seven faces with emotional expressions, four faces were presented in the central row of the matrix, and the remaining faces were presented in the central column of the top or bottom row of the matrix. In trials that included nine or eleven faces with emotional expressions, eight faces were presented in the central row and columns, and the remaining faces were presented in the corners of the matrix.

The faces were presented for 1,000 ms, as in Experiment 2. The sessions included seven conditions in which one, three, five, six, seven, nine, and 11 of the 12 faces presented had emotional expressions, and each condition was presented 20 times for the dense and distributed presentation patterns. Therefore, participants completed 560 trials. Numbers of faces with emotional expressions and presentation patterns were randomized. Participants completed 10 practice trials prior to the initiation of each session.

### Results

**Fig. 7:**
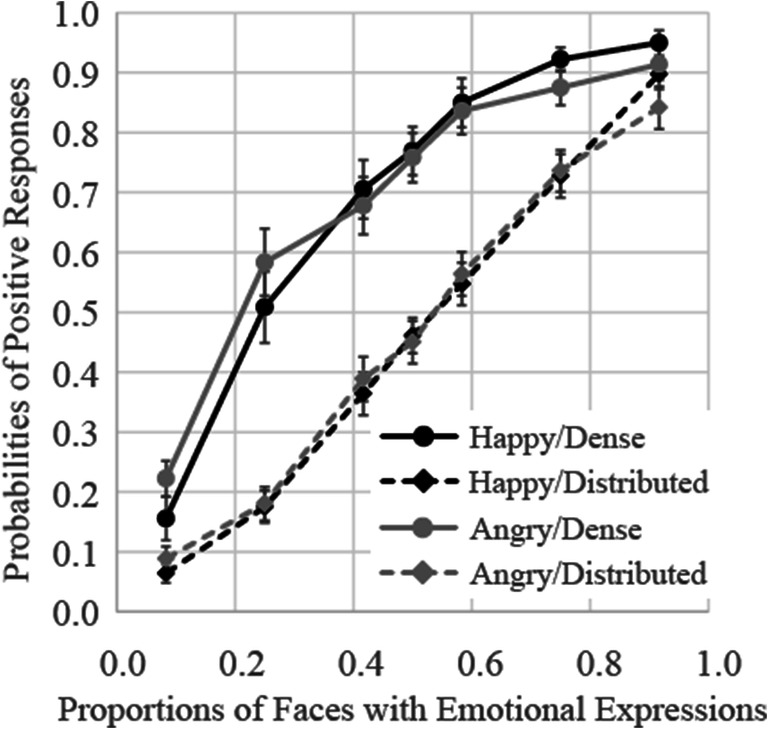
Probabilities of judgments indicating that faces with expressions were presented more frequently in Experiment 4. Error bars represent standard errors

The results for Experiment 4 are shown in Fig. 7. The function looks linear for the distributed presentation pattern, but gradually decreases in the amount of change (i.e., negatively accelerated) for the dense presentation pattern. The results of a three-way (presentation pattern × facial expression × proportion of emotional stimuli) ANOVA showed that the main effects of presentation pattern, and proportion of emotional stimuli, were both significant, *F*(1, 17) = 193.81, *p* < .0001, ɳ_p_^2^ = .92, and *F*(6, 102) = 218.72, *p* < .0001, ɳ_p_^2^ = .93, respectively. The interaction between presentation pattern and proportion of emotional stimuli was significant, *F*(6, 102) = 16.30, *p* < .0001, ɳ_p_^2^ = .49. Other interactions were not significant.

Follow-up analysis of the interaction between presentation pattern and proportion of emotional stimuli showed that probabilities of positive responses for the dense presentation patterns were significantly higher relative to those observed for the corresponding distributed presentation patterns, *F*s(1, 17) > 15.61, *p*s < .001, ɳ_p_^2^ > .48. Difference of probabilities between presentation patterns was stronger when three, five, six, seven, and nine faces expressed emotions (the effect sizes ɳ_p_^2^ were over .78) than when one and eleven faces expressed (the effect sizes ɳ_p_^2^ were .55 and .48, respectively).

The average PSEs and JNDs are shown in the rows of Experiment 4 of Table 1. The PSEs were 0.29 and 0.27 in the dense condition, indicating participants judged that the faces expressing anger or happiness were presented more frequently in groups for 50% of trials, even though just three or four faces expressed emotion. These results indicated that participants’ judgments were strongly weighted toward the small group of faces presented at the center of the pattern matrix. The results of two-way ANOVA (presentation pattern × facial expression) on the PSE showed a main effect of presentation pattern, *F*(1, 17) = 141.08, *p* < .0001, ɳ_p_^2^ = .89, indicating that the PSE in the dense pattern was lower than in the distributed pattern. However, a main effect of facial expression and an interaction between presentation pattern and facial expression were not significant, *F*(1, 17) = 0.04, *p* = .85, ɳ_p_^2^ = .002, *F*(1, 17) = 0.45, *p* = .51, ɳ_p_^2^ = .03, respectively. Two-way ANOVA on the JNDs also showed a main effect of presentation pattern, *F*(1, 17) = 7.83, *p* = .01, ɳ_p_^2^ = .32, indicating that the JND in the dense pattern was lower than in the distributed pattern. Neither main effects nor interaction were not significant, *F*s(1, 17) < 2.54, *p* > .13, ɳ_p_^2^ < .13.

### Discussion

The results of Experiment 4 showed that participants’ judgments as to which expression was presented more frequently differed between the two presentation patterns. If their judgments were based on probability calculated using the entire ensemble, the results would not have differed between presentation patterns, as the expected values calculated using the entire ensemble were the same. However, the PSE and JND in the dense patterns was much smaller than in the distributed patterns, suggesting that judgments as to which expression was presented more frequently were based on a small group of faces.

For the distributed presentation pattern, the results were identical to the presentation patterns used in Experiments 1 and 2. Since the two presentation patterns were randomized within the same blocks in Experiment 4, it was unlikely that participants employed different strategies in accordance with the presentation patterns.

## Experiment 5

Participants strongly weighted a small number of faces in their judgments in Experiment 4, in which the size of the stimuli was the same as that described for previous experiments. One explanation is that the participants’ calculation of face ensembles was limited to a smaller area than the entire presentation area of the faces. Experiment 5 sought to determine whether participants used subsampling of a small area or a small number of faces. Therefore, the size of the stimuli was reduced to a quarter of the size of the stimuli in Experiment 4 (i.e., height and width were halved). If the weighting were based on area, we would expect them to be able to perceive ensembles with more faces with such a stimulus size.

### Procedures

The task was the same as that described for Experiment 4, apart from the sizes of the faces and their presentation area (i.e., 2.0° wide × 2.4° high for each face and 8.2° wide × 7.1° high for the presentation area).

### Results

**Fig. 8:**
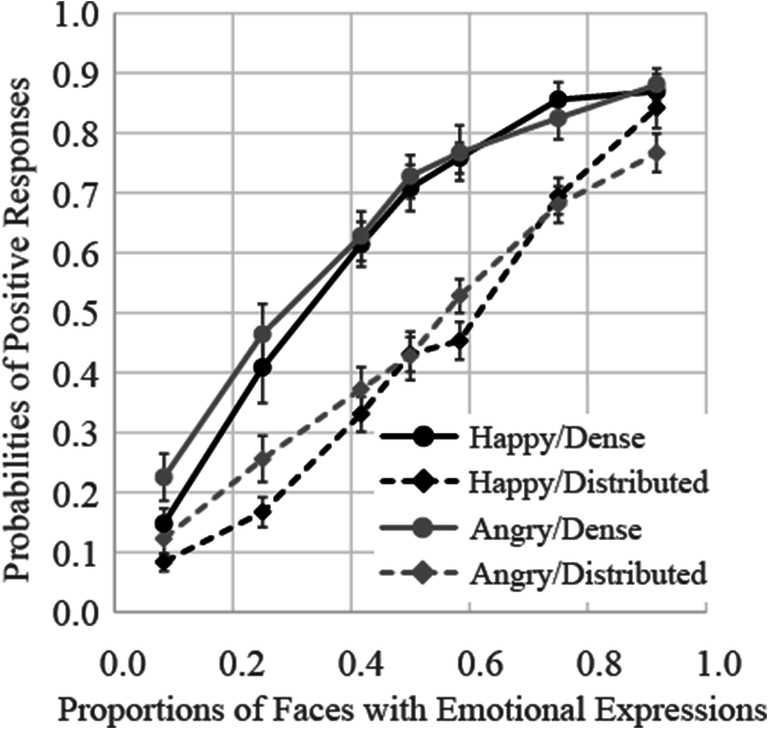
Probabilities of judgments indicating that faces with emotional expressions were presented more frequently in Experiment 5. Error bars represent standard errors

The results of Experiment 5 are shown in Fig. 8. The results of a three-way (presentation pattern × facial expression ×proportion of emotional stimuli) ANOVA showed that the main effects of presentation pattern and proportion of emotional stimuli were both significant, *F*(1, 17) = 128.69, *p* < .0001, ɳ_p_^2^ = .88 and *F*(6, 102) = 222.20, *p* < .0001, ɳ_p_^2^ = .93, respectively. Furthermore, the interactions between presentation pattern and proportion of emotional stimuli, and between facial expression and proportion of emotional stimuli, were both significant, *F*(6, 102) = 9.56, *p* < .0001, ɳ_p_^2^ = .36, and *F*(6, 102) = 2.96, *p* = .01, ɳ_p_^2^ = .15, respectively. The interactions between presentation pattern and facial expression and between presentation pattern, facial expression, and proportion of emotional stimuli were nonsignificant.

Follow-up analysis of the interaction between presentation pattern and proportion of emotional stimuli showed that the probabilities of judgments indicating that faces with emotional expressions were presented more frequently in the dense presentation patterns were significantly higher relative to those observed for the corresponding distributed presentation patterns in all proportion emotional stimuli conditions, *F*s(1, 17) > 9.50, *p*s < .007, ɳ_p_^2^ > .36. However, as shown in Experiment 4, difference of judgment probabilities between presentation patterns was not strong with one and eleven faces expressing emotion (the effect sizes ɳ_p_^2^ were .41 and .36, respectively) compared with other conditions (the effect sizes ɳ_p_^2^ were over .54).

Follow-up analysis of the interaction between facial expression and proportion of emotional stimuli showed that the proportion of angry faces was overestimated more frequently, relative to that of happy faces, with only one face expressing emotion (*p* = .03, ɳ_p_^2^ = .24).

The average PSEs and JNDs are shown in the rows of Experiment 5 of Table 1. The PSEs were 0.35 and 0.32 in the dense condition, indicating participants again judged that the faces expressing anger or happiness were presented more frequently in groups on 50% of trials, even though approximately four of the faces expressed emotion. These results indicated that participants’ judgments were strongly weighted toward a small number of faces rather than a small are of the ensemble. The results of two-way ANOVA on the PSE showed a main effect of presentation pattern, *F*(1, 17) = 138.58, *p* < .0001, ɳ_p_^2^ = .89, but a main effect of facial expression and an interaction between presentation pattern and facial expression were not significant, *F*s(1, 17) < 1.46, *p*s > .24, ɳ_p_^2^ < .08. Two-way ANOVA on the JND also showed a main effect of presentation pattern, *F*(1, 17) = 5.44, *p* = .03, ɳ_p_^2^ = .24, indicating that the JND in the dense pattern was lower than in the distributed pattern, but neither main effects nor interaction were not significant, *F*s(1, 17) < 2.83, *p*s > .11, ɳ_p_^2^ < .14.

### Discussion

In Experiment 5, the results replicated the tendency observed in Experiment 4, regardless of stimulus size. To compare the results of Experiments 4 and 5, a four-way (experiment × presentation pattern × facial expression ×proportion of emotional stimuli) ANOVA on the probability of positive responses was conducted. The results showed neither a significant main effect of the experiment, *F*(1, 34) = 2.64, *p* = .11, ŋ_p_^2^ = .07 nor interactions including the experiment, *F*s < 2.39, *p*s > .13, ŋ_p_^2^ < .07. Therefore, perception of ensembles of facial expressions was weighted towards a small number of faces rather than a small area.

## Experiment 6

**Fig. 9:**
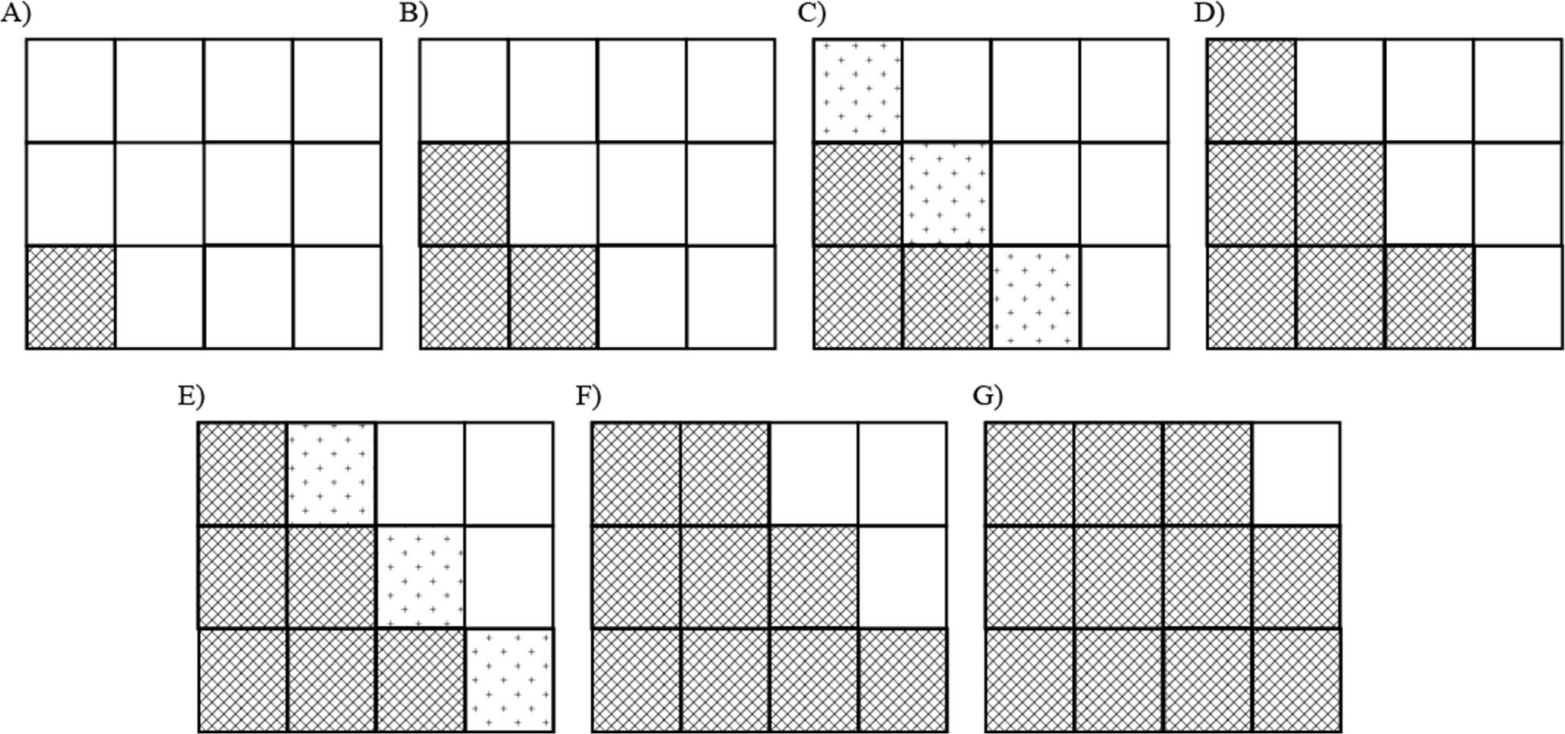
Schema for the dense presentation patterns at the bottom left in Experiment 6. **a**, **b**, **c**, **d**, **e**, **f**, and **g** indicate that one, three, five, six, seven, nine, and eleven faces expressing emotion were presented, respectively. The cells with diagonal lines indicate that faces were always presented in each pattern, whereas the cells filled with plus signs indicate that faces were presented randomly in those locations. When faces with emotion were dense at the top left, top right, and bottom right, the illustrated presentation pattern was vertically, orthogonally, and horizontally reflected, respectively

In Experiments 4 and 5, faces with emotional expressions were dense at the center of the presentation matrix. To examine whether dense emotional faces, even if not in the center, could capture and bias the observer’s attention, emotional faces were randomly dense at one of the corners of the presentation matrix every trial. If a group of emotional faces capture the participant’s attention, the results would be the same as those in Experiments 4 and 5. Otherwise, if observers calculate ensembles based on faces in the center of the presentation matrix regardless of the location of a group of emotional faces, the results would indicate smaller PSE and JND in the dense condition. This is because faces in the center do not express emotions when smaller numbers of faces express emotions (i.e., they are clustered near to the corner of the presentation matrix), whereas all faces around the center always express emotions when larger numbers of faces express emotions (see Fig. 9).

### Procedure

The task was the same as that described for Experiment 4, but in the dense presentation pattern, the presentation of faces with emotional expressions was dense at one of the corners of the presentation matrix. Figure 9 shows a schema when faces with emotional expressions were dense at the bottom left.

The faces were presented for 1,000 ms. The number of trials was the same as Experiment 4. Therefore, participants completed 560 trials. Numbers of faces with emotional expressions and presentation patterns were randomized.

### Results

**Fig. 10:**
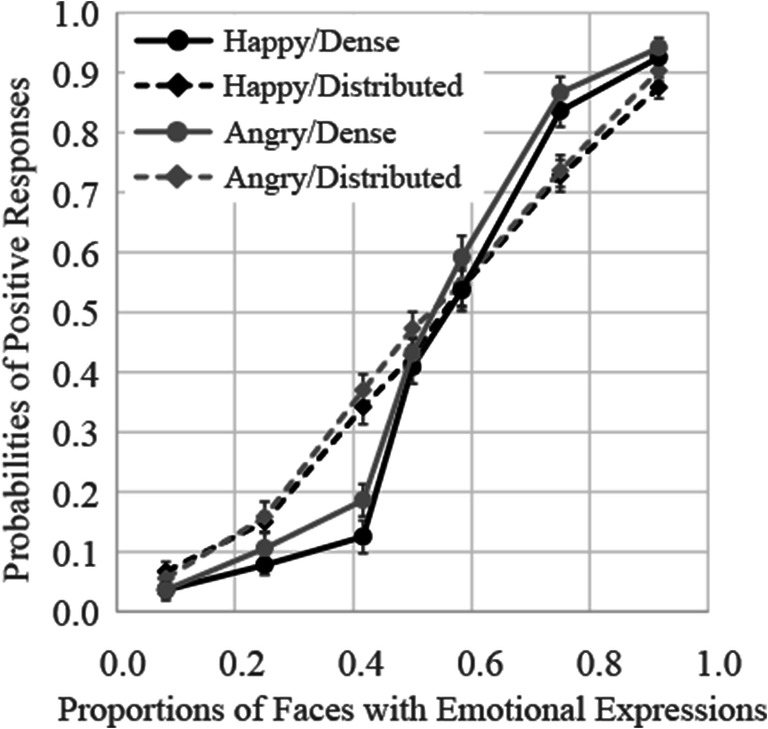
Probabilities of judgments indicating that faces with expressions were presented more frequently in Experiment 6. Error bars represent standard errors

The results for Experiment 6 are shown in Fig. 10. The results of a three-way (presentation pattern × facial expression ×proportion of emotional stimuli) ANOVA showed that the main effect of proportion of emotional stimuli was significant, *F*(6, 102) = 591.18, *p* < .0001, ɳ_p_^2^ = .97, but the main effects of presentation pattern, and facial expression were not significant, *F*(1, 17) = 2.52, *p* = .13, ɳ_p_^2^ = .13, *F*(1, 17) = 2.08, *p* = .17, ɳ_p_^2^ = .11, respectively. The interaction between presentation pattern and proportion of emotional stimuli was significant, *F*(6, 102) = 21.85, *p* < .0001, ɳ_p_^2^ = .56. Other interactions were not significant.

Follow-up analysis of the interaction between presentation pattern and proportion of emotional stimuli showed that probabilities of positive responses for the dense presentation patterns with one, three, and five faces expressing emotions were significantly lower relative to those observed for the corresponding distributed presentation patterns, *F*s(1, 17) > 7.28, *p*s < .02, ɳ_p_^2^ > .30. However, probabilities of positive responses for the dense presentation patterns with nine and eleven faces expressing emotions were significantly higher than the corresponding distributed presentation patterns, *F*s(1, 17) > 6.32, *p*s < .02, ɳ_p_^2^ > .27. No differences in probabilities of positive responses were observed with six and seven faces expressing emotions, *F*s(1, 17) < 1.02, *p*s > .33, ɳ_p_^2^ < .06. These results indicated that participants’ judgments were based on the small group of faces presented at the center of the pattern matrix because they rarely judge that faces with emotional expressions were more frequent when faces in the center did not express emotions (i.e., one, three, and five faces showed expressions in the dense presentation patterns) whereas they did when faces in the center showed expressions (i.e., nine and eleven faces showed expressions in the dense presentation patterns).

The average PSEs and JNDs are shown in the rows of Experiment 6 of Table 1. The PSEs were close to 0.50, and the results of two-way ANOVA on the PSE showed neither main effects nor interaction, *F*s(1, 17) < 1.08, *p*s > .32, ɳ_p_^2^ < .06. On the other hand, two-way ANOVA on the JND showed a main effect of presentation pattern, *F*(1, 17) = 76.04, *p* < .0001, ɳ_p_^2^ = .82, but a main effect of facial expression and an interaction between presentation pattern and facial expression were not significant, *F*s(1, 17) < 0.20, *p* > .66, ɳ_p_^2^ < .01.

### Discussion

The results of Experiment 6 were qualitatively different from those of Experiments 4 and 5; that is, participants rarely judged that faces with emotional expressions were more frequent when one, three, and five faces showed expressions in the dense presentation patterns, whereas they mostly judge that emotional faces were more frequent when nine and eleven faces showed expressions. There were no differences in positive responses when six and seven faces expressed emotions. If their judgments were based on dense emotional faces, probabilities of positive responses for the dense presentation patterns would be significantly higher relative to the corresponding distributed presentation patterns even when smaller numbers of faces express emotions as Experiments 4 and 5 showed. The results in Experiment 6 indicated that participants’ judgments were highly weighted by the small group of faces presented at the center of the crowd.

To compare the results of Experiments 4 and 6, a four-way (experiment × presentation pattern × facial expression ×proportion of emotional stimuli) ANOVA on probabilities of positive responses was conducted. The results showed a significant three-way interaction of experiment × presentation pattern × proportion of emotional stimuli, *F*(6, 204) = 28.30, *p* < .0001, ŋ_p_^2^ = .45, indicating that difference in positive responses between presentation patterns differs between experiments.

## Experiment 7

In Experiments 1–6, faces were presented with the hair and neck included, and the results showed that people could not perceive ensembles of facial expressions within the entire group of members. By contrast, faces were presented with the hair and neck cropped out in some previous studies (e.g., Bai et al., 2015; Haberman, Lee, & Whitney, 2015b; Haberman & Whitney, 2010). A parsimonious explanation is that it is more difficult to extract facial information from faces with the hair and neck than without them. Therefore, to investigate the robustness of finding in this study, we presented faces without the hair and neck and asked participants to determine which of two facial expressions was presented more frequently within a group.

### Procedures

**Fig. 11:**
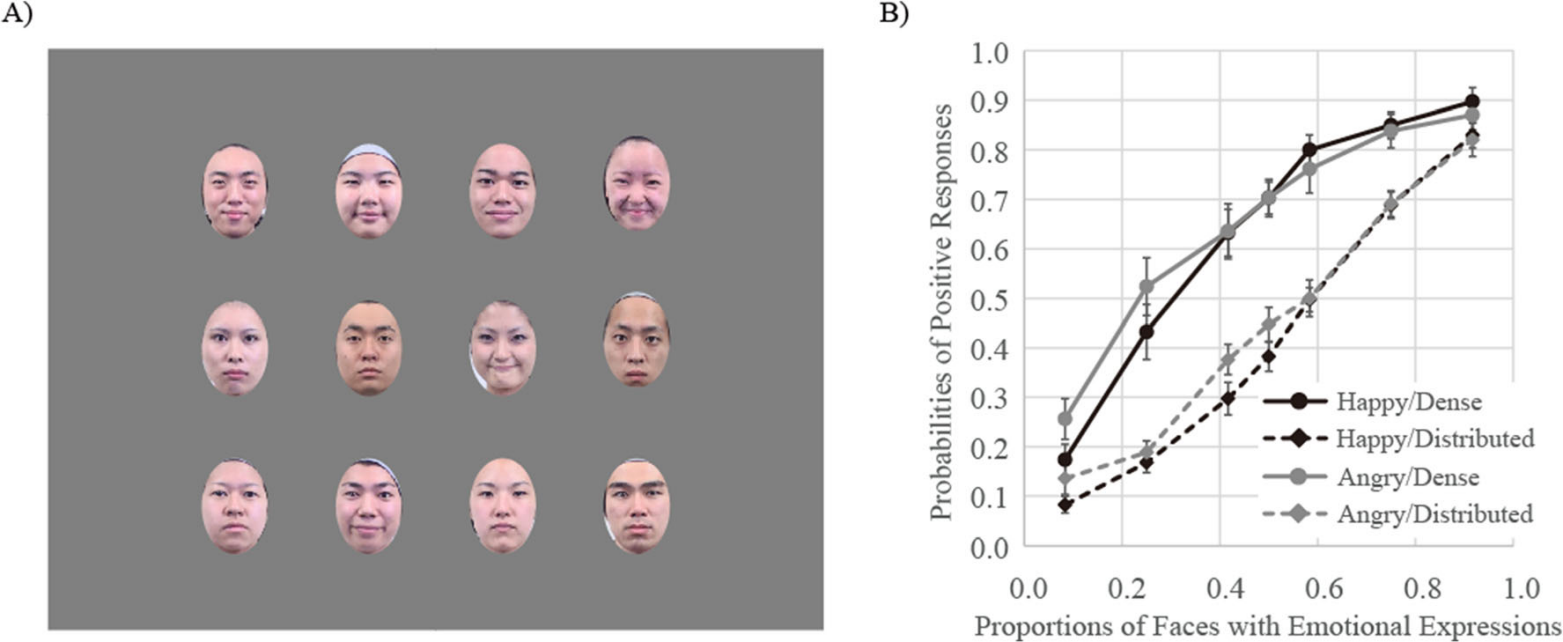
An example of stimuli used in Experiment 7 (**a**), and the probabilities of judgments indicating that faces with emotional expressions were presented more frequently in Experiment 7 (**b**). Error bars represent standard errors.

The task was the same as that described for Experiment 4, but the hair and neck were cropped from faces (see Fig. 11a).

### Results

The results of Experiment 7 are shown in Fig. 11b. The pattern was the same as in Experiments 4–6. The results of a three-way (presentation pattern × facial expression ×proportion of emotional stimuli) ANOVA showed that the main effects of presentation pattern and proportion of emotional stimuli were both significant, *F*(1, 16) = 101.41, *p* < .0001, ɳ_p_^2^ = .86 and *F*(6, 96) = 173.44 *p* < .0001, ɳ_p_^2^ = .92, respectively. Furthermore, the interaction between presentation pattern and proportion of emotional stimuli was significant, *F*(6, 96) = 14.78, *p* < .0001, ɳ_p_^2^ = .48. Other main effects and interactions were nonsignificant.

Follow-up analysis showed that probabilities of positive responses for the dense presentation patterns were significantly higher relative to those observed for the corresponding distributed presentation patterns, *F*s(1, 16) > 12.17, *p*s < .003, ɳ_p_^2^ > .43.

The average PSEs and JNDs are shown in the rows of Experiment 7 of Table 1. The PSEs were 0.33 in the dense condition, indicating participants again judged that the faces expressing anger or happiness were presented more frequently in groups on 50% of trials, even though approximately four of the faces expressed emotion. The results of two-way ANOVA on the PSE showed a main effect of presentation pattern, *F*(1, 16) = 73.04, *p* < .0001, ɳ_p_^2^ = .82, but a main effect of facial expression and an interaction between presentation pattern and facial expression were not significant, *F*s(1, 16) < 0.16, *p* > .69, ɳ_p_^2^ < .05. Two-way ANOVA on the JND showed neither main effects nor interaction, *F*s(1, 16) < 3.26, *p* > .09, ɳ_p_^2^ < .17.

### Discussion

Experiment 7 showed again different probabilities of positive responses between two presentation patterns and a smaller PSE for the dense presentation pattern than the distributed presentation pattern. Although the JND was also smaller for the dense presentation pattern than the distributed presentation pattern, it did not reach statistical significance (*p* = .09). These results suggest that trend in judgments as to which expression was presented more frequently were weighted towards a small group of faces even when faces did not contain the hair and neck.

## Experiment 8

Finally, we investigated individual differences in majority judgments of facial expressions in the crowd. Yang et al. (2013) showed that the PSE related with social anxiety when happy and angry faces were simultaneously presented. Focusing on the number of faces calculated for statistical summary perception, we examined relationships between the performance of this and visual working memory (VWM) in this experiment. VWM is associated with attentional control and executive function (Adam et al., 2015; Hiebel & Zimmer, 2015), and a previous study suggests that attentional control accounts for ensemble performance (Myczek & Simon, 2008). The perception of statistical summary of complicated objects such as facial expression could also be associated with this function.

In this experiment, we used the same procedure in Experiment 4 to measure the accuracy of perception for distribution. Therefore, in addition to investigating the relationship between this and VWM, we also examined whether the results of Experiment 4 could be replicated.

### Procedure

The majority estimation task was the same as that described for Experiment 4. In addition to the majority estimation task, participants performed the change detection task developed by Luck and Vogel (1997). In this task, an encoding display consisting of 1–8 color patches were simultaneously presented, and participants judged whether a following display (i.e., probe) presented after the retention period was the same as the encoding display (50% of trials) or if one of the color patches changed (50% of trials). The presentation duration of the encoding display was 100 ms and the retention period was 900 ms. The probe display was presented until participants responded. Trials with 1–8 color patches were repeated 24 times. Therefore, the total number of trials in the change detection task was 192.

### Results

**Fig. 12:**
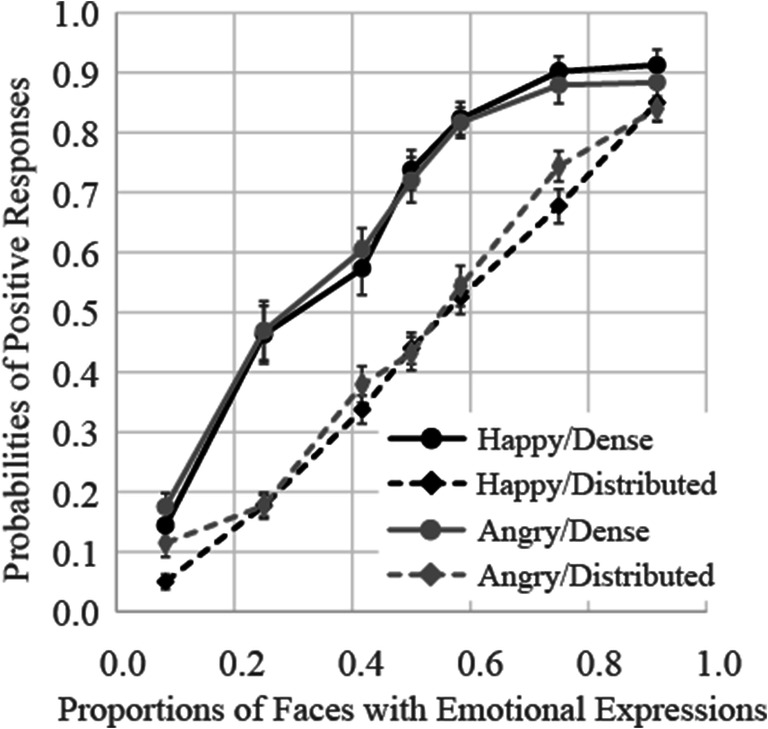
Probabilities of judgments indicating that faces with expressions were presented more frequently in Experiment 8. Error bars represent standard errors

For the majority judgment task, the results shown in Fig. 12 were consistent with those in Experiment 4. The results of a three-way (presentation pattern × facial expression ×proportion of emotional stimuli) ANOVA showed that the main effects of presentation pattern and proportion of emotional stimuli were both significant, *F*(1, 23) = 110.04, *p* < .0001, ɳ_p_^2^ = .83, and *F*(6, 138) = 254.15, *p* < .0001, ɳ_p_^2^ = .92, respectively. The interaction between presentation pattern and proportion of emotional stimuli was significant, *F*(6, 138) = 21.93, *p* < .0001, ɳ_p_^2^ = .49. Other interactions were not significant. Follow-up analysis of the interaction between presentation pattern and proportion of emotional stimuli showed that probabilities of positive responses for the dense presentation patterns were significantly higher than the distributed presentation patterns in all proportion conditions, *F*s(1, 23) > 23.66, *p*s < .0001, ɳ_p_^2^ > .51 (the effect sizes ɳ_p_^2^ were larger than .61 when the three to nine faces expressed emotions, while they were relatively small, .51, when the one and eleven faces expressed emotions).

The average PSEs and JNDs are shown in the rows of Experiment 8 of Table 1. The PSEs were 0.34 and 0.33 in the dense condition. The results of two-way ANOVAs on the PSE and JND showed that only main effects of presentation pattern on both the PSE and JND was significant, *F*(1, 23) = 103.67, *p* < .0001, ɳ_p_^2^ = .82, and *F*(1, 23) = 9.49, *p* = .005, ɳ_p_^2^ = .29. These suggest that the results in Experiment 4 can be replicated in this experiment.

For the change detection task, Pashler’s Ks was calculated as indices of the capacity of VWM (Pashler, 1988)^3^. Accuracy was relatively high (more than 88%) when the number of color patches to be memorized were one, two, and three (congruent with the results of Luck & Vogel, 1997), and moreover, Pashler’s Ks were stable when color patches were presented for more than three. Therefore, we averaged Pashler’s Ks with 4–8 color patches and employed it as an index of VWM capacity of the participant.

**Fig. 13:**
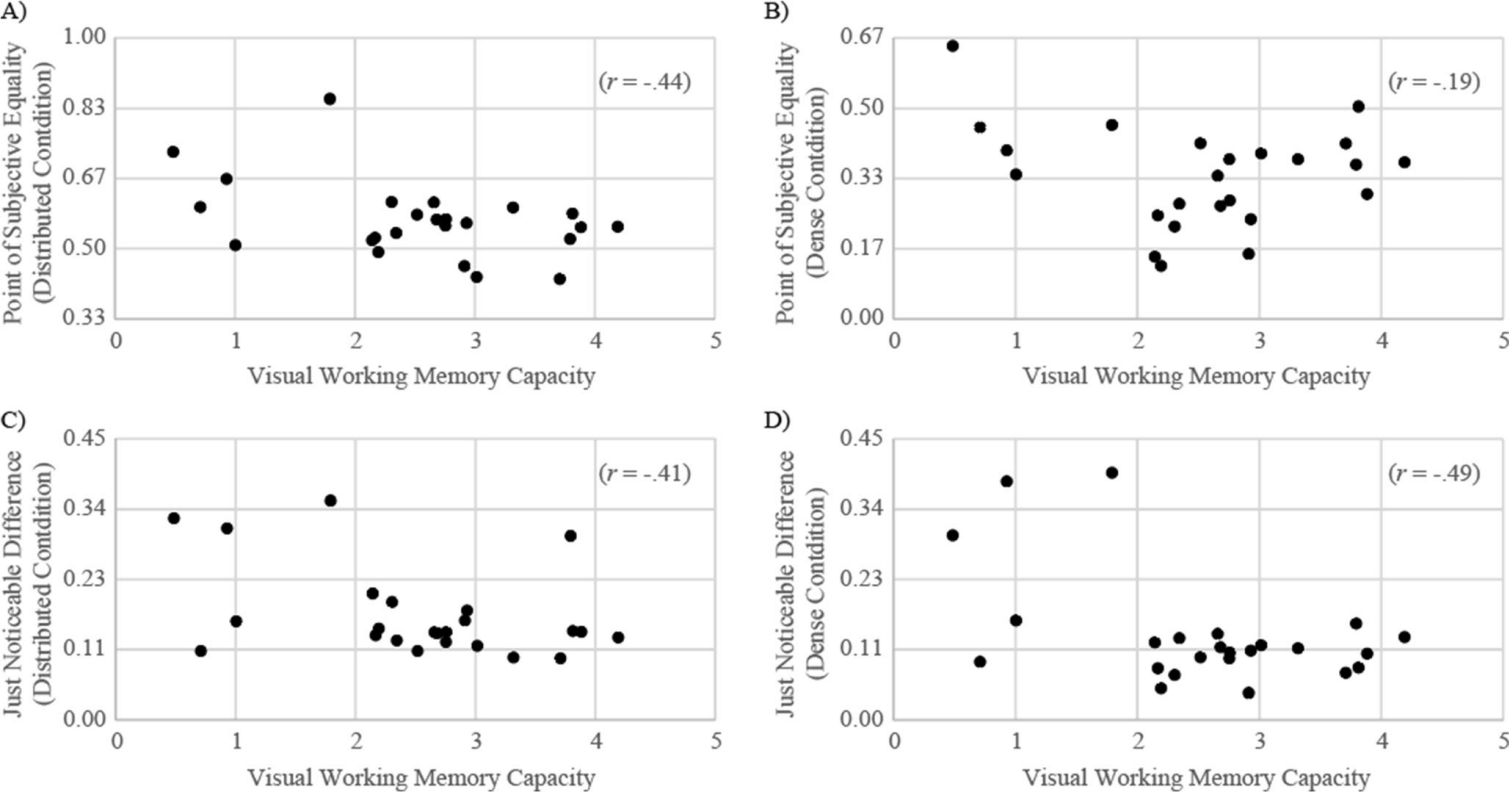
Scatter plots describe the relationships between the PSE and VWM in the distributed condition (**a**) and in the dense condition (**b**), and between the JND and VWM in the distributed condition (**c**) and in the dense condition (**d**) in Experiment 9. Each dot represents an individual participant

The scatter plots shown in Fig. 13 describe the relationships between the PSE and the index of VWM, and between the JND and the index of VWM. The correlation was significantly negative between the PSE and VWM in the distributed condition (*r* = -.44, *p* = .031), whereas it was not significant in the dense condition (*r* = -.19, *p* = .36). Moreover, the correlation was significantly negative between the JND and VWM both in the distributed condition (*r* = -.41, *p* = .049) and in the dense condition (*r* = -.49, *p* = .015)^4^.

### Discussion

Experiment 8 replicated the results of Experiments 4–7; that is, participants’ judgments as to which expression was presented more frequently differed between the two presentation patterns.

Moreover, Experiment 8 demonstrated a moderate negative relationship between the PSE in the distributed condition and VWM capacity. Considering that the PSEs in the distributed condition were scattered around 0.5 for individuals with higher VWM capacity, the results indicated that the higher VWM capacity individuals have, the less biased they got in this condition. Compared with this, individuals who have lower VWM capacity did not judge that emotional faces were the majority until the proportion rate became comparatively high. Interestingly, this tendency was not observed in the dense condition, suggesting that people tend to perform majority judgments of facial expressions based on the center of the crowd regardless of visual working memory capacity when they were collected there. Moreover, negative correlations between the JND and VWM capacity suggest that the higher VWM capacity participants have, the more their summary perception is precise.

## General Discussion

This study showed three main findings. First, instantaneous judgments as to which facial expression was presented more frequently were imprecise when large numbers of real distinctive faces were presented. Even when the hair and neck were cropped from faces, participants could not use the distribution information from a whole face, suggesting that it is hard to perceive facial expression ensemble extracted from many realistic faces. Second, instantaneous ensemble perception of realistic facial expressions highly weight towards a small number of faces in the center of crowd rather than all the faces. This result is consistent with the previous studies conducted by Ji et al. (2014). Moreover, this study found that this processing was independent of stimulus size. Third, the moderate negative relationships between the PSE and VWM capacity and between the JND and VWM capacity were obtained. Specifically, these relationships were obvious in the distributed presentation pattern (i.e., facial expressions were randomly mixed in the presentation matrix), suggesting that ensemble perception and working memory share processing or are deeply related with each other.

Facial expression is determined by combinations of facial muscles (described as action units by Ekman & Friesen, 1978). For example, a happy face is characterized by tightened muscles around the eyes, with the cheeks and corners of the lips raised; whereas an angry face is characterized by lowered, drawn-together eyebrows with tensed and raised eyelids and lip-pressed-against-lip mouth (Ekman & Friesen, 2003). Failure to perceive ensembles of realistic facial expressions could indicate difficulty in perceiving ensembles of multiple feature-binding objects. Some studies have suggested that faces and facial expressions are recognized holistically, rather than in separate parts (e.g., Calder et al., 2000), indicating that if each expression is processed holistically and summarized as a specific parameter (e.g., whether expression is happiness or not), ensembles of faces could be perceived as easily as ensembles of lower features. However, participants’ failure to perceive ensembles of facial expressions in the current study indicated that they would not represent each facial expression in this manner to understand a collective facial expression (or group mood).

The results of this study do not necessarily deny the previous results using morphed faces (merging multiple faces, e.g., de Fockert & Wolfenstein, 2008; Haberman & Whitney, 2007; Haberman & Whitney, 2009). This is because it is easier to calculate the average of emotional faces using one of the physical parameters of features in faces (i.e., the degree of raised lip corner) than using real distinctive faces. In the same way, another study showed that simple head rotation and gaze direction could be summarized in 16 faces (Florey et al., 2016). These findings suggest that individuals might perceive ensembles of individual features, even in faces.

Moreover, previous research showing perceive ensembles of facial expressions presented only four faces (Haberman, Brady, & Alvarez, 2015a; Haberman & Whitney, 2007, 2009), which is less than the current study; therefore, participants could have perceived ensembles with the faces presented in these studies. In addition to the difference in the numbers of faces presented between these previous studies and the current study, the durations of presentation in the previous studies (i.e., 2,000 ms) were longer relative to those used in the current study (i.e., 500 or 1,000 ms). The results of the current study did not refute the possibility that people could perceive ensembles of higher numbers of faces over longer periods, even if they are real distinctive faces. If observers are allowed sufficient time, they could either perceive ensembles of numerous faces simultaneously, or they could use a subsample of a small number of faces repeatedly, allowing them to eventually perceive ensembles of entire faces. These possibilities appear to be consistent with daily behavior, such as that involving the delivery of a speech to an audience.

On the other hand, the experiments conducted in this study could not fully rule out that participants perceive statistical summary extracted from all the group members. Some studies showed that faces in the central/foveal location would have larger weights in averaging (Florey et al., 2016; Ji et al., 2014), and attended items would also have larger weights than the unattended one (de Fockert & Marchant, 2008). Considering these studies, it might be possible that participants extracted distribution of items with different weights depending on location in the visual (or attentional) field. The results can exclude possibilities that they did not equally use all the face information to extract ensembles, and that emotional faces collectively presented in peripheral vision distort judgments automatically and instantly. Rather, the results support that judgement based on mainly some of faces in the central location, at least when they perform instantaneous judgment (i.e., this is a definition of ensemble perception), regardless of presentation sizes.

The PSEs and JNDs in a series of experiments in this study are summarized in Table 1. The PSEs in the dense presentation pattern of Experiments 4–8 are approximately between 0.25 and 0.35 (except for Experiment 6 because emotional faces were not presented in the center), indicating that participants estimated that the faces with emotional expressions were presented more frequently in 50% only when three or four emotional faces were presented in the central row. This number reminds us of the involvement of two cognitive functions to ensemble perception. The first of these functions is subitizing, which involves instantaneous judgment of a small number (i.e., three or four) of objects (Kaufman et al., 1949). The second function involves capacity for VWM (Luck & Vogel, 1997). Experiment 8 showed that the latter related to the JND, not the PSE, in the dense presentation pattern. Moreover, even in a relatively high proportion rate in the dense presentation pattern, participants’ performance kept improving little by little. Considered together, one speculative explanation of ensemble perception of distribution is that when distinctive items are presented as in this study, observers largely weight information presented in the central vision based on subitizing (or function in common with this), and complementarily use information around them depending on their capacity of VWM (e.g., without interfering with each other). This is just a proposal and speculative. In the future, further empirical investigations should be undergone (for example, other statistical summary perceptions except for majority judgments are according with this).

The proportion of angry faces were estimated higher than those of happy faces in Experiments 1 and 2. These results could have occurred because of the anger superiority effect examined in studies conducted by Eastwood et al. (2001) and Fox et al. (2000), which showed that angry faces captured attention more rapidly relative to happy faces, and that observers experienced greater difficulty in disengaging from angry faces compared to happy faces. Rapid capture of attention and difficulty disengaging from angry faces could cause observers to focus on angry faces and prevent them from shifting their attention to other faces. As observers rely on information based on only some of the faces, they might estimate the proportion of angry faces higher than happy faces in instantaneous judgments. Participants tended to overestimate the proportions of angry faces when they were few in number in the distributed condition of Experiments 5, and 7, but this was subtle in that condition of Experiments 4, 6, and 8. Moreover, they were not statistically significant except for Experiments 1, 2, and 5. Therefore, the relationship between the anger superiority effect and higher angry face estimation should be carefully examined in future studies.

We investigated whether participants could perceive distribution of all facial expressions when many real distinctive faces were presented. If participants recognized it instantaneously, we would expect them to correctly identify the more frequently presented expression within groups. However, they could not perform this judgment with precision, meaning that they could not use distribution information from a whole face. Considering subsampling due to limited-capacity processes, two presentation patterns of faces (dense and distributed patterns) were presented to participants, and they demonstrated that perception of ensembles of facial expressions was based on some, rather than all, of the faces. Moreover, individual differences in precision of statistical summary perception related to their visual working memory function.

## Appendix

If participants subsampled four faces from all the faces, the probability that the three facial expressions presented were subsampled can be calculated. The probability that all three facial expressions were subsampled is calculated as follows:

$$ \frac{{}_3{C}_3\times {}_9{C}_1}{{}_{12}{C}_4}=\frac{1}{55} $$

The probability that two of three facial expressions were subsampled is calculated as follows:

$$ \frac{{}_3{C}_2\times {}_9{C}_2}{{}_{12}{C}_4}=\frac{12}{55} $$

The probability that one of three facial expressions was subsampled is calculated as follows:

$$ \frac{{}_3{C}_1\times {}_9{C}_3}{{}_{12}{C}_4}=\frac{28}{55} $$

The probability that no facial expressions were subsampled is calculated as follows:

$$ \frac{{}_9{C}_4}{{}_{12}{C}_4}=\frac{14}{55} $$

Therefore, if participants’ responses were based on the probability of facial expressions from subsampled faces, the expected probability that faces with emotional expressions were presented more frequently is calculated as follows:

$$ \frac{1}{55}\times \frac{3}{4}+\frac{12}{55}\times \frac{1}{2}+\frac{28}{55}\times \frac{1}{4}=\frac{1}{4} $$

This expected value is the same as the proportion of faces with emotional expressions in the groups of faces (i.e., in this case, three of 12 faces), suggesting that the expected values of judgments based on probability using entire faces and subsampled faces are the same. Moreover, these relationships between expected values do not change in accordance with the size of subsamples (i.e., even if participants subsample six faces using entire faces, the expected probability that faces with emotional expressions are presented more frequently is $\frac{1}{4}$ when three facial expressions are presented).

## Footnotes

1 JND is defined as the 1/2 strength of difference between stimulus with a 25% probability of being judged a 75% probability of being judged. When fitting the cumulative Gaussian function, it can be converted to JND by multiplying the SD of the fitted Gaussian function by 0.675. Therefore, SD = 0.49 for the cumulative Gaussian function in previous studies can be interpreted as JND = 0.33 (meaning 0.49 × 0.675).

2 If perception was perfect, it would be expected that the results should be fitted to a step-function (i.e., positive response is 0 unless proportion of faces with emotional expression is under .50, and it reaches 1 after proportion is over .50) rather than sigmoid (c.f., PSE was .50 and JND was 0). However, since noises should be considered in the psychological experiments, we proposed a sigmoid-shape instead of a step-function.

3 Pashler’s K was calculated using this formulation: N*(h-f)/(1-f), in which N indicates the number of color patches to be memorized, h indicates hit rate, and f indicates false alarm rate. See more details in Pashler (1988).

4 Even if we employ overall average of Pashler’s Ks across all color patch conditions (1–8), the results were consistent. In this case, the correlation coefficients between the PSE and VWM in the distributed condition was *r* = -.40, *p* = .055, and in the dense condition was *r* = -.18, *p* = .41. The correlation between the JND and VWM in the distributed condition was *r* = -.43, *p* = .034, and in the dense condition was *r* = -.44, *p* = .031.
